# Supplementary material for: Identifying repeat domains in large genomes
Source: Genome Biol. 2006 Jan 31;7(1):R7. doi: 10.1186/gb-2006-7-1-r7 (PMC1431705; doi:10.1186/gb-2006-7-1-r7)
Supplement: Additional File 1 — A zipped file of browsable HTML files with a complete list of the connected components in the repeat domain graph of human Repbase. [file gb-2006-7-1-r7-S1.gz › html/subgraphs/890.pdf]

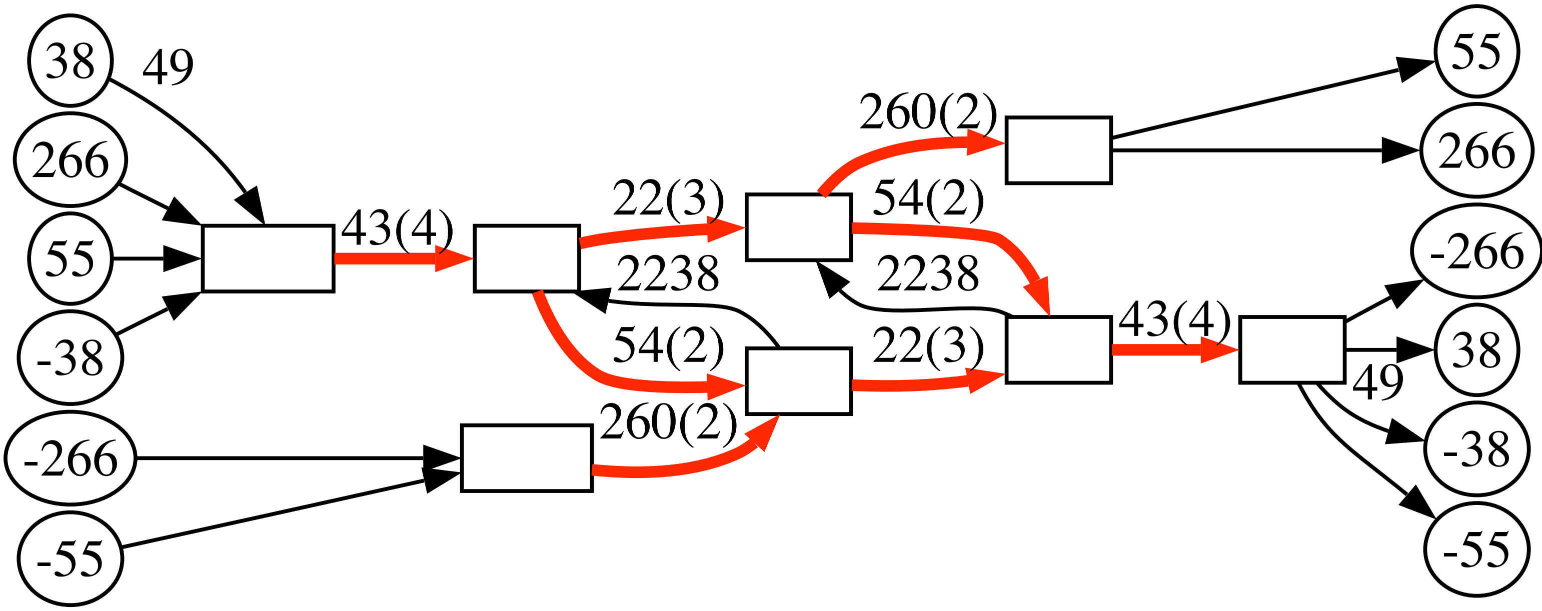
38
49
266
55
-38
-266
-55
43(4)
22(3)
2238
54(2)
260(2)
260(2)
54(2)
2238
22(3)
43(4)
55
266
-266
38
49
-38
-55
